# Role of Neutrophils on the Ocular Surface

**DOI:** 10.3390/ijms221910386

**Published:** 2021-09-27

**Authors:** Yongseok Mun, Jin Sun Hwang, Young Joo Shin

**Affiliations:** 1 Department of Ophthalmology, Hallym University Medical Center, Hallym University College of Medicine, Seoul 07442, Korea; yongseokmun@hallym.or.kr (Y.M.); hotsayme@naver.com (J.S.H.); 2 Hallym BioEyeTech Research Center, Hallym University College of Medicine, Seoul 07442, Korea

**Keywords:** ocular surface disease, dry eye syndrome, neutrophil, neutrophil extracellular trap

## Abstract

The ocular surface is a gateway that contacts the outside and receives stimulation from the outside. The corneal innate immune system is composed of many types of cells, including epithelial cells, fibroblasts, natural killer cells, macrophages, neutrophils, dendritic cells, mast cells, basophils, eosinophils, mucin, and lysozyme. Neutrophil infiltration and degranulation occur on the ocular surface. Degranulation, neutrophil extracellular traps formation, called NETosis, and autophagy in neutrophils are involved in the pathogenesis of ocular surface diseases. It is necessary to understand the role of neutrophils on the ocular surface. Furthermore, there is a need for research on therapeutic agents targeting neutrophils and neutrophil extracellular trap formation for ocular surface diseases.

## 1. Introduction

The ocular surface of the eyeball is the part of the eye in contact with the outside world, serving as a primary barrier against external substances and pathogens [1]. The cornea is a transparent tissue that refracts light entering the eye, focusing it on the retina and acting as a barrier against the outside [2]. The conjunctiva is a mucous membrane that attaches to the cornea and becomes the surface surrounding the eyeball [3]. It forms a conjunctival sac surrounding the inner eyelid and connecting to the eyelid [2]. In addition, the conjunctiva is connected to the nasal mucosa and supplied with tears from the lacrimal gland through the lacrimal duct [2]. The mucous membrane of the conjunctiva has many blood vessels and produces a large amount of mucus from goblet cells [3]. The subconjunctival tissue contains many lymphoid tissues and the immune system [3]. The ocular surface immune system can be divided into innate and adaptive immune systems [4]. The innate immune system includes basophils, dendritic cells, eosinophils, Langerhans cells, mast cells, monocytes and macrophages, neutrophils, and natural killer cells, whereas the adaptive immune system includes T and B lymphocytes [5].

Neutrophils are members of the innate immune system and are at the forefront against infection, but they are involved in adaptive immunity through interactions with T and B cells [6]. They have been reported to play an essential role in autoimmune diseases such as rheumatoid arthritis, systemic lupus erythematosus, and anti-neutrophil cytoplasmic antibodies-associated vasculitis (AAV) [7]. Although the ocular surface is in contact with external pathogens, both innate and adaptive immunity are involved in the pathogenesis of dry eye syndrome, characterized by tear instability and inflammation of the ocular surface [8]. Therefore, the role of neutrophils in the ocular surface is discussed in this article.

## 2. Methods

A systematic literature search was performed on PubMed and Medline for papers published before 30 August 2021. The following combined search terms were used: “neutrophil,” “neutrophil extracellular traps,” “dry eye,” “ocular surface,” and “NETosis.” Both human and animal studies were included in the outcome evaluation. Correspondences, notes, and editorials were excluded. Neither language filter nor limitation of publication time was applied during the literature search. References of the retrieved studies were also reviewed manually to identify relevant articles.

**Figure 1:**
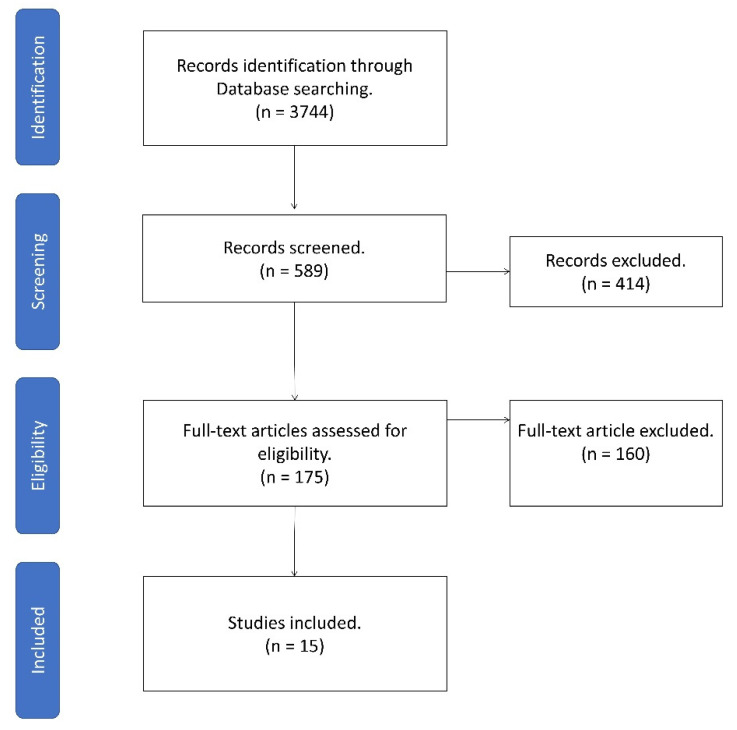
Flowchart of the study screening process for original studies in neutrophils for the ocular surface.

A review of the literature in the PubMed database identified 175 articles. After extensive study, 15 articles were included (Figure 1).

## 3. Neutrophils in Immunity 

Neutrophils are considered short-term and terminally differentiated phagocytes with no significant gene expression or regulatory role in adaptive immunity [6]. However, in recent years, opinions on the role of neutrophils have been developing. Neutrophils are primarily short-term polymorphonuclear cells (PMNs) related with the first line combatant to pathogens, which can phagocytose potentially harmful antigens and trigger strong antimicrobial defenses, including the release of reactive oxygen species (ROS) such as superoxide and neutrophil extracellular traps [9]. Neutrophils extrude their nucleus or mitochondrial DNA to form neutrophil extracellular traps, called “beneficial suicide” [10]. In addition, neutrophils can act as the myeloid-derived suppressor cells (MDSCs) to inhibit adaptive T cell functions such as the expansion of T cells, the activation of other immune cells, or the secretion of cytokines [11].

In response to pathogen exposure, the promptest action of neutrophils is phagocytosis, which arises within minutes [12]. Phagocytosis and opsonization are primarily associated with the ingestion of small microbes, while large-sized bacteria and fungi induce the extrusion of granules from the neutrophils [13]. The primary and secondary granules, which contain cationic defensins, myeloperoxidase (MPO), neutrophil elastase, iron chelators, lactoferrin, human neutrophil lipocalin, and several metalloproteinases, are released primarily in regards to harsh sterile or infectious stimuli, releasing adenosine monophosphate (AMP) and proteases that can efficiently break down bacterial and fungal proteins [14]. Tertiary granules comprising matrix metalloproteinases and gelatinases are released to facilitate their migration through the extracellular matrix [15,16].

Neutrophil extracellular traps have been described as one of the ways in which neutrophils remove microbes [17,18]. Neutrophils release a chromatin network adorned with granular-derived antibacterial peptides and active enzymes, including cathepsin G, MPO, and neutrophil elastase [19]. Neutrophil extracellular trap formation, known as NETosis, was initially reported as an extracellular antibacterial form resisting microbe. However, recently it has been reported that neutrophil extracellular trap formation is involved in autoimmune/rheumatic and auto-inflammatory disease states beyond microbial death [20]. Neutrophil extracellular traps are released after infection with Gram (+) and (-) bacteria, especially by large-sized microbes [21]. Neutrophils generally kill small microbes through phagocytosis, but larger microbes that are not easily digested release cytoplasmic granules and promote nuclear translocation of neutrophil elastases to form neutrophil extracellular traps [17]. In addition, neutrophil extracellular trap formation is induced by nitric oxide, autoantibodies, cytokines such as interleukin (IL)-1β, IL-6, IL-8, and tumor necrosis factor (TNF)-α, hydrogen peroxide, lipopolysaccharides, phorbol-12-myristate-13-acetate, ionophores for calcium ion, and the interaction with activated platelets or vascular endothelial cells [22,23].

The three main signaling pathways of neutrophil extracellular trap formation have been discussed. First, after phorbol-12-myristate-13-acetate stimulates neutrophils through the protein kinase C (PKC) and Raf-mitogen-activated protein kinase (MEK) extracellular signal-regulated kinase signaling pathways, it induces the activation of nicotinamide adenine dinucleotide phosphate oxidase 2, triggering the associated signaling cascade and neutrophil extracellular trap formation through the production of ROS [20,24]. ROS formation promotes the migration of two key enzymes, MPO and neutrophil elastase, stored in the neutrophil granules, to the nucleus and induces chromatin decondensation, leading to the release of nuclear neutrophil extracellular traps [20]. Hydrogen peroxide is converted to hypochlorous acid by MPO, which activates neutrophil elastase to break down the cytoskeleton and nuclear membrane, allowing neutrophil extracellular trap excretion [25]. Second, the increase in intracellular calcium levels activates the peptidylarginine deiminase 4 (PAD4) enzyme, which moves to the nucleus, leading to histone citrullination and chromatin decondensation [26]. This mechanism is independent of nicotinamide adenine dinucleotide phosphate oxidase 2 [27]. Third, another form of neutrophil extracellular trap formation is the mitoNET formation [28]. Mitochondria are degraded and release the oxidized mitochondrial DNA into the extracellular space by mitochondrial ROS production or the stimulation of toll-like receptor 4 or complement factor 5a receptor [28,29]. Neutrophil extracellular trap formation induced by nitric oxide and phorbol myristate acetate induces both nuclear and mitochondrial neutrophil extracellular trap formation [23]. 

Neutrophil extracellular trap is thought to be enrolled in the onset of autoimmune and autoinflammatory diseases [30]. Autoantibodies to neutrophil extracellular trap components, including the citrullinated histones with DNA, MPO-DNA complexes, and neutrophil elastase-DNA complexes, are common in several systemic autoimmune diseases [31]. Defects in the process of neutrophil extracellular trap formation, excessive neutrophil extracellular trap formation, and delayed neutrophil extracellular trap formation clearance are all associated with autoimmunity [31]. Neutrophil extracellular traps have been suggested to play a pivotal role in various autoimmune diseases, including systemic lupus erythematosus, vasculitis, rheumatoid arthritis, and chronic inflammatory bowel diseases such as Crohn’s disease and ulcerative colitis [32,33,34,35]. Circulating and synovial neutrophils in patients with rheumatoid arthritis are more prone to forming neutrophil extracellular traps than in healthy controls [36,37]. Neutrophil extracellular trap formation is a source of autoantibody and stimulates inflammatory responses in rheumatoid arthritis [37]. In rheumatoid arthritis, anti-citrullinated protein antibodies are formed, associated with neutrophil extracellular trap formation and neutrophil count [38]. Neutrophil extracellular trap formation can also be provoked by neutrophil binding of anti-neutrophil cytoplasmic antibodies and anti-ribonuclear protein (RNP) antibodies [39]. As an extracellular bactericidal mechanism used by neutrophils, neutrophil extracellular traps go through steps that include ROS production, PAD4 activation, granule formation, chromatin decondensation, and active release of DNA/histone/cathelicidin antimicrobial peptide cocktail into the extracellular space [27]. 

Peptidyl-arginine deiminase 2 (PAD2) and PAD4 are the posttranslational modification enzymes converting protein arginine or mono-methylarginine to citrulline [40]. PAD2 and PAD4 are implicated in the pathogenesis of several autoimmune diseases [41]. Histone citrullination by PAD2 and PAD4 is essential for neutrophil extracellular trap formation [31,42,43]. Hypercitrullination in synovial fluid and anti-citrullinated protein antibodies in plasma are found in rheumatoid arthritis [44], suggesting that the hypercitrullinated molecules may serve as autoantigen. PAD2 and PAD4 are potential biomarkers and therapeutic targets of sepsis [45]. PAD4 inhibitor block neutrophil extracellular trap formation [46], reducing bleomycin fibrosis [47,48]. Simultaneous inhibition of PAD2 and PAD4 ameliorates neutrophil extracellular trap formation and reduces inflammatory cytokine production [49]. 

**Figure 2:**
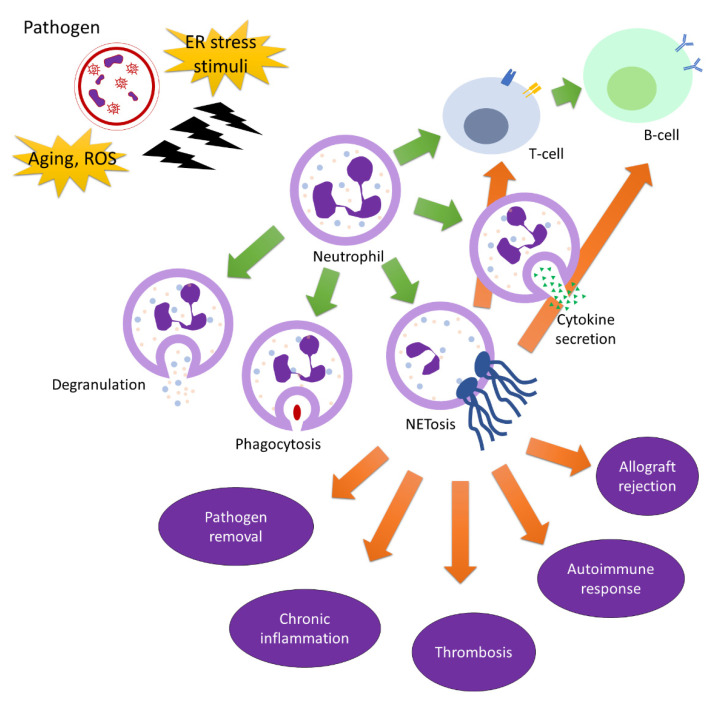
Neutrophils in immunity.

Neutrophil elastase is a proteolytic enzyme belonging to the chymotrypsin-like family of serine-proteolytic enzymes, a protein packaged in cytoplasmic neutrophil granules of neutrophil granulocytes [50]. Neutrophil elastase is unnecessary for neutrophil extracellular trap formation with non-infectious stimuli [51], but degrades the extracellular matrix including elastin, collagens, proteoglycan, fibronectin, immunoglobulins, and surfactant proteins and stimulates the pro-inflammatory cytokines to contribute to inflammation [52]. Neutrophil elastase reduces the secretion of secretory leukoproteinase inhibitor (SLPI) by lung epithelial cells [53]. The role of neutrophils in the immune system is summarized in Table 1 and Figure 2.

## 4. Neutrophils in Adaptive Immunity 

Neutrophils have been suggested to modulate adaptive immunity, although they have been thought to be a significant member of innate immunity [65]. They regulate T cell proliferation and cytokine production [66,67]. Neutrophil extracellular trap activates dendritic cells, causing Th1 polarization to produce cytokines from T cells [67]. Neutrophils directly regulate T cells by engaging with antigen-presenting cells [68,69,70] and suppress the cytotoxic activity of innate and adaptive killer cells in cancer [71]. Further, they enhance the responsiveness of CD8+ T cells to T-cell receptor triggering signals [54], whereas neutrophils from common variable immunodeficiency patients actively inhibit T cell activation and secretion of IFN-γ via the ROS formation [55]. Contact between T cells and neutrophil extracellular trap enhances T cell responses to specific antigens [72]. Programmed cell death protein 1 (PD-1) axis, expressed on the surface of activated T-cells promoting apoptosis, blocks neutrophil cytotoxicity in cancer [73]. T cells enhance neutrophil function in host resistance in candida infection [74]. The T cells promote phagocytosis and chemotaxis of neutrophils through C-C motif chemokine ligand 8 [75], whereas the B cells secrete antibodies in response to antigen [76]. Neutrophils help B cell activation to produce antibodies in the spleen through IL-10 and IL-21 [56,57] and destruct the pathogens by opsonization [77,78]. Interaction between neutrophils and B cells leads to B cell differentiation and activation and neutrophil infiltration through C-C motif chemokine ligand 1/C-C motif chemokine ligand 2 [79]. Furthermore, the allergic response has been described to be associated with the role of neutrophils [58]. Elevation of neutrophil-attracting chemokine IL-8 and IL-17 was found in allergic disease, and neutrophils were infiltrated into the tissues in a toll-like receptor 4-, myeloid differentiation protein-2-, and C-X-C motif chemokine receptor 2-dependent manner to sensitize the allergic response [58,59]. Activated neutrophil promotes T cell activation in allergic disease [59] and contributes to IgE production in allergen-specific B cells through presenting antigen [60]. 

During immunologic rejection of organ transplantation, neutrophils are the first immune cells to infiltrate in the transplanted organs [80]. Neutrophils are essential in promoting alloimmune responses and immunological rejection is ameliorated by inhibiting neutrophil extracellular traps [61,62,63]. However, deficient neutrophil extracellular trap formation has been reported in patients undergoing bone marrow transplantation [64]. 

## 5. Role of Autophagy in Neutrophils

Autophagy is a critical mechanism in cell biology that allows cells to maintain nutrient and energy homeostasis by removing damaged or harmful intracellular components and is involved in cell survival and death depending on cell type and stress conditions [81]. Autophagy with impaired control has been linked to various diseases, including neurodegenerative diseases, inflammatory diseases, and cancer [82]. During autophagy, cytoplasmic components are surrounded by double-membrane vesicles known as autophagosomes, delivered to lysosomes for degradation (autologous lysosomes) [83]. In this process, damaged cellular elements or intracellular pathogens are detected and removed to protect cells and nourish them by recycling cytoplasmic macromolecules and organelles [83].

Autophagy is required to regulate inflammation by modulating pathogen removal, antigen presentation, cytokine production, and immune response and is a regulator of neutrophil function [84]. Autophagy is required to develop long-term survival subsets of neutrophil-derived granulocytes positive for CD15, CD66b, CD63, CD11b, MPO, and neutrophil elastase [85]. Autophagy is an important modulator of neutrophil extracellular trap formation through mTOR-dependent pathways [86]. Autophagy positively modulates neutrophil extracellular trap formation, and thus, diminishing autophagy is associated with decreased neutrophil extracellular trap formation [87]. Autophagy activation through the inhibition of the mammalian target of rapamycin (mTOR) by rapamycin promotes neutrophil extracellular trap formation, whereas autophagy inhibition by wortmannin suppresses neutrophil extracellular traps release [87]. Phosphoinositide 3-kinases (PI3K)–AKT–mTOR axis links autophagy and neutrophil extracellular trap induction and significantly impacts both processes [88]. In infection with invasive bacteria, autophagy in neutrophils precedes neutrophil extracellular trap formation, and autophagy-related 5 knockdown blocks neutrophil extracellular trap formation [89]. In infection with invasive *E. coli*, autophagy in neutrophils precedes neutrophil extracellular trap formation, and autophagy-related 5 silencing completely blocks neutrophil extracellular trap formation [89]. Neutrophils isolated from aged mice defective in autophagy-related 5 showed a reduction of neutrophil extracellular trap release [90]. 

In recent years, the role of autophagy in neutrophil-mediated inflammation and autoimmune diseases has been described [91]. Neutrophils move to the site of inflammation as the frontline of innate immunity [92]. Autophagy is a protective mechanism for neutrophil-mediated inflammation, and inhibiting autophagy can lead to uncontrolled inflammation [93]. Autophagy inhibits degranulation and affects nicotinamide adenine dinucleotide phosphate oxidase-mediated ROS production, down-regulating apoptosis and affecting neutrophil tissue invasion [94]. Knockdown of autophagy-related 5 and autophagy-related 7 reduces the inflammatory function of neutrophils by inhibiting ROS production and degranulation [95]. In the inflammatory process, endoplasmic reticulum stress can provoke neutrophil autophagy, and autophagy can suppress endoplasmic reticulum stress [96,97]. Inhibition of autophagy through the knockdown of nucleotide oligomerization domain (NOD)-like receptor pyrin domain-containing protein 3 (NLRP3) or inhibition of NLRP3 inflammasome promotes neutrophil recruitment and phagocytosis, thereby enhancing pathogen removal and improving the survival of septic mice [98]. 

Autophagy is required in the production and extrusion of several neutrophil cytokines [87,99]. The release of IL-1β, one of the pro-inflammatory cytokines secreted from neutrophils, was suppressed by autophagy inhibitors or autophagy-related 5 silencing. Inhibition of autophagy may be an effective treatment in neutrophil-mediated inflammatory diseases [35].

## 6. Endoplasmic Reticulum Stress in Neutrophils

Endoplasmic reticulum stress is involved in the pathogenesis of many diseases such as dry eye, rheumatoid arthritis, diabetes, dementia, and cancers [100,101,102,103]. It is linked to cellular dysfunction, inflammation, oxidative stress, apoptosis, and autophagy. Mitochondrial activity and endoplasmic reticulum stress are required for neutrophil differentiation [104]. Endoplasmic reticulum stress reduces during both neutrophil and macrophage differentiations, and the activities of protein kinase R-like endoplasmic reticulum kinase and activating transcription factor 6 were decreased, and that of inositol-requiring enzyme 1-α is enhanced during neutrophil differentiation [104]. The role of endoplasmic reticulum stress of neutrophils was investigated in acute lung injury [105]. Elevated endoplasmic reticulum stress levels were observed in infiltrated neutrophils in the acute lung injury mice model [105]. Sensors for endoplasmic reticulum stress, including protein kinase R-like endoplasmic reticulum kinase, activating transcription factor 6, and inositol-requiring kinase 1, were enhanced in neutrophil in acute lung injury [105]. Suppression of endoplasmic reticulum stress inhibited the inflammation [105]. Inositol-requiring enzyme 1-α is a crucial regulator of neutrophil extracellular traps through ROS generation and caspase-2 activation [106]. Endoplasmic reticulum calcium level is increased in the neutrophils in cystic fibrosis in response to endoplasmic reticulum stress response, which exaggerates the inflammation [107]. Tunicamycin-induced endoplasmic reticulum stress signaling (protein kinase R-like endoplasmic reticulum kinase/activating transcription factor 4/CCAAT-enhancer-binding protein homologous protein signaling) aggravates airway inflammation via elevation of inflammatory cytokines (IL-6, IL-8, and TNF-α) in a murine model of neutrophilic asthma [108]. Endoplasmic reticulum stress/X-box-binding protein 1 enhances mucin secretion through the influence of neutrophil elastase [109]. Neutrophil induces apoptosis in cancer cells through an endoplasmic reticulum stress pathway [110]. 

## 7. Neutrophils in Aging 

Neutrophil extracellular traps remove the old vessels to promote remodeling [111]. However, aging drives neutrophils to be pathogenic, contributing to vascular diseases [112,113]. The intestinal microtome regulates neutrophil aging by enhancing C-X-C motif chemokine receptor 4 and reducing L-selectin [114]. Interaction between neutrophils and the microbiome contributes to the maturation of the immune system and the pathogenesis of immune-mediated diseases and cardiovascular diseases [115,116]. Aged neutrophils are characterized by altered expression of surface molecules such as lymphocyte function-associated antigen-1, macrophage-1 antigen, toll-like receptor-4, platelet endothelial cell adhesion molecule-1, and higher oxidative stress levels [117]. In addition, neutrophil extracellular traps are more prone to be formed in aged neutrophils [117]. Neutrophil extracellular traps accumulation compromises organ functions and impairs revascularization and vascular repair after ischemic injuries [118]. Delayed clearance of neutrophil extracellular traps facilitates autoimmune reactivity [119]. 

Neutrophil aging induces chronic inflammation of vessels, affecting lacrimal glands and ocular surfaces. Since neutrophil extracellular trap formation is also easily activated on the ocular surface in the elderly, it may be one of the pathogenic mechanisms of dry eyes in elderly patients.

## 8. Human Factors Affecting Neutrophils

Smoking has been reported to elevate the neutrophil count in blood [120,121] and to increase neutrophil elastase-induced inflammation through the elevation of IL-8 production and proteinase-activated receptor-2 [122]. Neutrophils are stimulated to produce C-X-C chemokine ligand 8 through toll-like receptor-9 receptor activation, inducing chronic inflammation [123]. Air pollution, including particulate matter 2.5 (PM2.5) and particulate matter 10 (PM10), causes inflammation, where neutrophils are involved [124,125]. Smoking enhances the impairment of neutrophil function by air pollution [126]. Chronic alcohol drinking impairs normal neutrophil extracellular trap formation and phagocytosis [127,128], although single alcohol drinking exaggerates the neutrophil response to the microbiome [129]. Neutrophil activation and functions are suppressed by alcohol consumption through C-X-C chemokine ligand 1/C-X-C chemokine receptor type 2 [130]. Vitamin D has been reported to have an immunomodulatory function [131]. Vitamin D enhances the production of IL-8 in neutrophils, although it does not affect the neutrophil phagocytic capacity in response to lipopolysaccharide [132]. Vitamin D deficiency is associated with high blood neutrophil count and neutrophil reactive oxygen species levels [133,134]. 

Hyperlipidemia is associated with blood neutrophil count [135] and increases leukotriene B4 production in neutrophils by increasing the nuclear translocation of 5-lipoxygenase, which initiates the synthesis of leukotrienes from arachidonic acid [136]. Atherosclerosis is facilitated by hyperlipidemia-induced elevation of blood neutrophil count [137]. In diabetes, high blood glucose level affects the metabolism of neutrophils [138] and enhances transient neutrophil activation followed by the inhibition of cell activity [139]. Hyperglycemic condition primes neutrophils to produce more superoxide and cytokines and to form more neutrophil extracellular traps than in normoglycemic conditions [140].

## 9. Neutrophils in COVID-19

Coronavirus disease (COVID-19) is an infectious disease which is caused by the severe acute respiratory syndrome coronavirus 2 (SARS-CoV-2) [141,142] and involves the lungs accompanying the associated systemic complications [143,144]. In COVID-19, neutrophils are heavily infiltrated into tissues and play an important role in the pathogenesis of complications [145]. SARS-CoV-2 directly stimulates neutrophil extracellular trap formation [146], inducing epithelial cell death [146] and circulates in the blood vessels to contribute to immunothrombosis by secreting IL-1α and cathepsin G [147,148]. The presence of circulating neutrophil extracellular traps may be a prognostic factor for COVID-19 because they block blood vessels and increase mortality [144,149,150,151]. Targeting neutrophil extracellular trap in COVID-19 may improve the prognosis and reduce the complications by preventing neutrophil extracellular trap-induced thrombosis [152]. 

## 10. Neutrophils on the Ocular Surface

Neutrophil extracellular trap formation by neutrophils has been reported to protect against corneal infection by Pseudomonas and Aspergillus on the ocular surface [153,154,155]. Infectious keratitis is a severe disease that can threaten vision. Neutrophils kill the pathogen by phagocytosis, degranulation, and neutrophil extracellular trap formation as the front line against pathogen [156,157,158]. In *Pseudomonas keratitis*, neutrophils infiltrate and form neutrophil extracellular traps to kill the pathogens [159], but it can cause corneal damage [160]. Killing Pseudomonas with inhibition of neutrophil extracellular traps may be a useful way to reduce corneal damage and improve clinical prognosis [160]. Bacterial biofilms are difficult to treat once they are formed, but in this particular situation, neutrophil extracellular traps confine the pathogen and prevent it from spreading to surrounding tissues [153]. Fungus, such as aspergillus or candida, is too large to be removed by phagocytosis, and thus, it is removed by neutrophil extracellular trap formation [161,162]. Inhibition of neutrophil extracellular trap exacerbates fungal keratitis [161]. Viral keratitis, such as herpes virus or adenovirus, is accompanied by infiltration of neutrophils [163,164,165] through secretion of cytokines or chemokines including IL-6, IL-17, or C-X-C motif chemokine ligand 1/keratinocytes-derived chemokine [165,166], which can lead to corneal damage [167]. In ocular surface burns, neutrophils first appear in the ocular tissue, remove the dead tissue, and trigger an inflammatory and fibrotic reaction [168]. Excessive neutrophil infiltration appears in severe eye burns and is known as an indicator of poor corneal prognosis [169]. Inhibition of neutrophil extracellular trap formation has been reported to increase the rate of corneal wound healing in burns through inhibition of nuclear factor kappa-light-chain-enhancer of activated B cells activation [170]. Dry eye disease is characterized by tear instability, hyperosmolarity, and ocular surface inflammation and is associated with ocular discomforts [171]. Neutrophil infiltration and degranulation occur in patients with dry eye disease [172,173]. Our previous study revealed that systemic endoplasmic reticulum stress induced the neutrophil infiltration in lacrimal glands, which provoked ocular surface inflammation in the dry eye model [97]. It has been reported that neutrophil extracellular trap formation markers, such as neutrophil elastase, MPO, and citrullinated histone H3, exist on the ocular surface [174]; extracellular DNA production and clearance mechanisms are dysregulated in dry eye disease, which results in ocular surface inflammation [175]. Hyperosmolarity promotes neutrophil extracellular trap formation, which was inhibited by anti-inflammatory/proapoptotic agents [176]. Meibomian glands dysfunction, which is associated with blepharitis, is a common cause of evaporative dry eye disease [177]. Neutrophil extracellular trap formation orchestrates the inflammation and occludes the ducts of exocrine glands and the blood vessels [178]. Neutrophil extracellular trap obstructs meibomian glands and cause meibomian glands duct dilation and acinar atrophy [179]. 

Neutrophils affect the ocular surface in ways other than neutrophil extracellular trap formation. Systemic immune-inflammation index (SII) levels, neutrophil-to-lymphocyte ratio, and platelet-to-lymphocyte ratio were higher in patients with dry eye disease [180]. The neutrophil-to-lymphocyte ratio is calculated as neutrophil count divided by the lymphocyte count [181] and may be useful to estimate the activity of autoimmune and inflammatory diseases [182]. Further, the neutrophil-to-lymphocyte ratio increases in patients with non-Sjögren dry eye disease [183], suggesting that non-Sjögren dry eye disease may be associated with systemic inflammation [183]. The platelet-to-lymphocyte ratio is a novel inflammatory marker, which may be used in many diseases for predicting inflammation and mortality [184].

**Figure 3:**
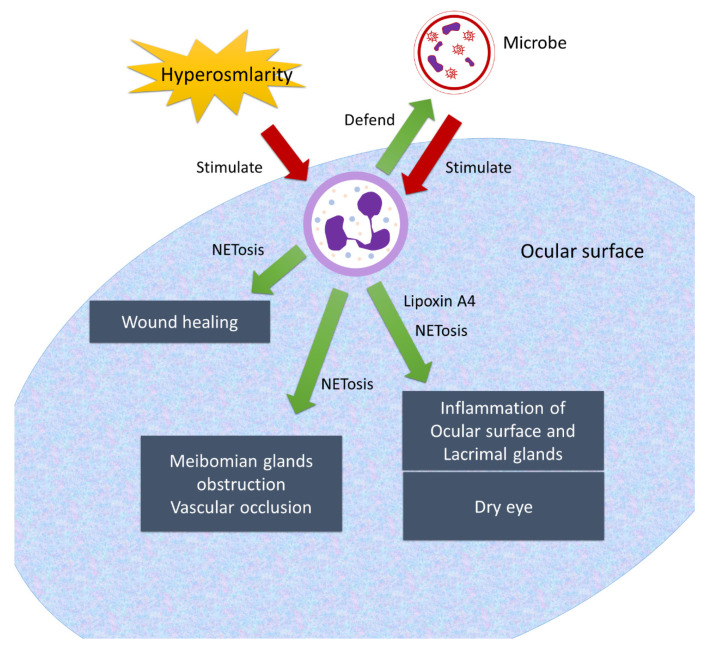
Neutrophils on the ocular surface.

In dry eye disease, the secretion of lipoxin A4 from neutrophils is regulated by dietary ω−3 docosahexaenoic acid (DHA) [185]. Elevated lipoxin A4 levels in ocular tissue contribute to the severity of dry eye disease by affecting Treg, TH1, and TH17 effector cells [186]. Sjögren’s syndrome (SS) is an autoimmune disease involving lacrimal and salivary glands [187]. Autoantibodies to Sjögren’s syndrome antigen B (SSB), a ribonucleoprotein, have been frequently reported in SS [188]. It is unclear what role neutrophils play in SS, but SSB activates mitogen-activated protein kinase (MAPK) pathway and nuclear factor kappa-light-chain-enhancer of activated B cells signaling to induce IL-8 release from neutrophils [189]. There is a need for research on therapeutic agents targeting neutrophils and neutrophil extracellular trap formation for ocular surface diseases. The role of neutrophils in dry eye disease is summarized in Table 2 and Figure 3.

## 11. Drug Development 

Inhibition of neutrophil extracellular trap formation may reduce the inflammation and inflammation-associated damages on the organ, which can serve as a treatment option for dry eye disease or other autoimmune diseases [190,191]. The neutrophil inhibitor has been reported to decrease neutrophil-mediated lung damage in patients with acute respiratory distress syndrome and suggested to modulate the tissue destruction and the disease course [192]. 

Several neutrophil elastase inhibitors have been developed. Sivelestat, a selective neutrophil elastase inhibitor, prevented phorbol myristate acetate-induced acute lung injury [193,194], enhanced coronary blood flow, and ameliorated myocardial damage after myocardial arrest [195]. Furthermore, it has shown its protective effect in neuromyelitis optica [196], refractory Kawasaki disease [197], knee osteoarthritis [198], steatohepatitis [199], and systemic inflammation such as burn [200]. BAY 85-8501, another selective and potent neutrophil elastase inhibitor, has been revealed to reduce pulmonary disease inflammation [201]. DX-890, a small-protein neutrophil elastase inhibitor, showed anti-inflammatory effects through reducing neutrophil trans-epithelial migration, releasing activity, and neutrophil elastase-induced cytokine expression in airway epithelial cells [52]. MPH-966, neutrophil elastase inhibitor, attenuated intestinal injury and ameliorated intestinal microbiome [202].

PAD2 inhibitor improved survival from endotoxemia induced by lipopolysaccharide through inhibiting neutrophil extracellular trap formation and secretion of pro-inflammatory cytokines [49,203]. PAD4 inhibitors regulate the neutrophils by preventing active nicotinamide adenine dinucleotide phosphate oxidase complex and an oxidative burst in neutrophils [204]. 

Neutrophil inhibition can be a promising treatment option in dry eye disease. Neutrophil extracellular trap formation inhibition by acetylsalicylic acid and dexamethasone promotes corneal epithelial cell migration in corneal alkali burns through modulating nuclear factor kappa-light-chain-enhancer of activated B cells signaling [170]. 

## 12. Conclusions

The ocular surface is a gateway that contacts the outside and receives stimulation from the outside. Neutrophil infiltration and degranulation occur on the ocular surface. Degranulation, phagocytosis, neutrophil extracellular trap formation, called NETosis, and autophagy in neutrophils are involved in the pathogenesis of ocular surface diseases. It is necessary to understand the role of neutrophils in the ocular surface. Furthermore, there is a need for research on therapeutic agents targeting neutrophils and neutrophil extracellular trap formation for ocular surface diseases.

## Figures and Tables

**Table 1:** Role of neutrophil in immunity.

References	Findings
Richards and Endres 2014. [12]	Phagocytosis of neutrophil against pathogen
Brinkmann et al. 2004, Brinkmann and Zychlinsky 2012, Keshari et al. 2012, Fonseca et al. 2018 [17,18]	Neutrophil extracellular trap formation of neutrophil against pathogen
Angelidou et al. 2018, Frizinsky et al. 2019, Tsourouktsoglou et al. 2020, Bach et al. 2020, Fatemi et al. 2021 [30,31,32,34,35]	Neutrophil extracellular trap formation contributing to autoimmune diseases
Liu et al. 2018, Li et al. 2020. [41,46]	PAD2/PAD4 activation in autoimmune diseases
Dunlevy et al. 2012, Martinod et al. 2016 [51,52]	Neutrophil elastase released from neutrophil causes inflammation
Puga et al. 2011, Cerutti et al. 2013, Governa et al. 2017, Vlkova et al., 2019 [54,55,56,57]	Interaction of neutrophil and adaptive immune response including T cells and B cells
Hosoki et al. 2016, Arebro et al. 2017, Polak et al. 2019 [58,59,60]	Neutrophils contribute to allergic response
Jones et al. 2010, Glenn et al. 2016, Yıldızet al. 2021, Liu et al. 2021 [61,62,63,64]	Neutrophils contribute to immunological rejection in transplanted organ

**Table 2:** Role of neutrophils on the ocular surface.

References	Mode of Action or Mechanism	Organ
Cho et al. 2019 [97]	Neutrophil inflammation	Lacrimal glands
Sonawane et al. 2012, Barliya et al. 2017, Mahajan et al. 2021 [174,175,179]	Neutrophil extracellular formation	Ocular surface and meibomian glands
Ozarslan et al. 2020 [180]	Increased neutrophil-to-lymphocyte ratio	Blood
Gao et al. 2018 [186]	Lipoxin A4 amplification	Ocular surface
Wan et al. 2020 [170]	Wound healing of cornea	Cornea and ocular surface
Tibrewal et al. 2014 [176]	Hyperosmolarity of tear film promotes neutrophil extracellular traps formation	Ocular surface

## Abbreviations

SS	Sjögren’s syndrome
TNF-α	Tumor necrosis factor-α
NETosis	Neutrophil extracellular trap formation
PAD2	Peptidyl-arginine deiminase 2
PAD4	Peptidyl-arginine deiminase 4
PMN	Polymorphonuclear cells
ROS	Reactive oxygen species
MDSCs	Myeloid-derived suppressor cells
MPO	Myeloperoxidase
AMP	A denosine monophosphate
NLRP3	Nucleotide oligomerization domain (NOD)-like receptor pyrin domain-containing protein 3

## References

[1] Agrahari V., Mandal A., Agrahari V., Trinh H.M., Joseph M., Ray A., Hadji H., Mitra R., Pal D., Mitra A.K. (2016). A comprehensive insight on ocular pharmacokinetics. Drug Deliv. Transl. Res..

[2] Sridhar M.S. (2018). Anatomy of cornea and ocular surface. Indian J. Ophthalmol..

[3] Hodges R.R., Dartt D.A. (2013). Tear film mucins: Front line defenders of the ocular surface; comparison with airway and gastrointestinal tract mucins. Exp. Eye Res..

[4] Galletti J.G., de Paiva C.S. (2021). The ocular surface immune system through the eyes of aging. Ocul. Surf..

[5] Sokol C.L., Luster A.D. (2015). The chemokine system in innate immunity. Cold Spring Harb. Perspect. Biol..

[6] Mocsai A. (2013). Diverse novel functions of neutrophils in immunity, inflammation, and beyond. J. Exp. Med..

[7] Navegantes K.C., de Souza Gomes R., Pereira P.A.T., Czaikoski P.G., Azevedo C.H.M., Monteiro M.C. (2017). Immune modulation of some Autoimmune Dis.eases: The critical role of macrophages and neutrophils in the innate and adaptive immunity. J. Transl. Med..

[8] Reyes J.L., Vannan D.T., Eksteen B., Avelar I.J., Rodriguez T., Gonzalez M.I., Mendoza A.V. (2018). Innate and Adaptive Cell Populations Driving Inflammation in Dry Eye Disease. Mediators Inflamm..

[9] Nguyen G.T., Green E.R., Mecsas J. (2017). Neutrophils to the ROScue: Mechanisms of NADPH Oxidase Activation and Bacterial Resistance. Front. Cell Infect. Microbiol..

[10] Mesa M.A., Vasquez G. (2013). NETosis. Autoimmune Dis..

[11] Ostrand-Rosenberg S., Fenselau C. (2018). Myeloid-Derived Suppressor Cells: Immune-Suppressive Cells That Impair Antitumor Immunity and Are Sculpted by Their Environment. J. Immunol..

[12] Richards D.M., Endres R.G. (2014). The mechanism of phagocytosis: Two stages of engulfment. Biophys. J..

[13] Kobayashi S.D., Malachowa N., DeLeo F.R. (2017). Influence of Microbes on Neutrophil Life and Death. Front. Cell Infect. Microbiol..

[14] Eichelberger K.R., Goldman W.E. (2020). Manipulating neutrophil degranulation as a bacterial virulence strategy. PLoS Pathog..

[15] Lin M., Jackson P., Tester A.M., Diaconu E., Overall C.M., Blalock J.E., Pearlman E. (2008). Matrix metalloproteinase-8 facilitates neutrophil migration through the corneal stromal matrix by collagen degradation and production of the chemotactic peptide Pro-Gly-Pro. Am. J. Pathol..

[16] Chakrabarti S., Zee J.M., Patel K.D. (2006). Regulation of matrix metalloproteinase-9 (MMP-9) in TNF-stimulated neutrophils: Novel pathways for tertiary granule release. J. Leukoc. Biol..

[17] Brinkmann V., Zychlinsky A. (2012). Neutrophil extracellular traps: Is immunity the second function of chromatin?. J. Cell Biol..

[18] Brinkmann V., Reichard U., Goosmann C., Fauler B., Uhlemann Y., Weiss D.S., Weinrauch Y., Zychlinsky A. (2004). Neutrophil extracellular traps kill bacteria. Science.

[19] Kaplan M.J., Radic M. (2012). Neutrophil extracellular traps: Double-edged swords of innate immunity. J. Immunol..

[20] Delgado-Rizo V., Martinez-Guzman M.A., Iniguez-Gutierrez L., Garcia-Orozco A., Alvarado-Navarro A., Fafutis-Morris M. (2017). Neutrophil Extracellular Traps and Its Implications in Inflammation: An Overview. Front. Immunol..

[21] Manda A., Pruchniak M.P., Arazna M., Demkow U.A. (2014). Neutrophil extracellular traps in physiology and pathology. Cent. Eur. J. Immunol..

[22] Keshari R.S., Jyoti A., Dubey M., Kothari N., Kohli M., Bogra J., Barthwal M.K., Dikshit M. (2012). Cytokines induced neutrophil extracellular traps formation: Implication for the inflammatory disease condition. PLoS ONE.

[23] Hoppenbrouwers T., Autar A.S.A., Sultan A.R., Abraham T.E., van Cappellen W.A., Houtsmuller A.B., van Wamel W.J.B., van Beusekom H.M.M., van Neck J.W., de Maat M.P.M. (2017). In vitro induction of NETosis: Comprehensive live imaging comparison and systematic review. PLoS ONE.

[24] Fonseca Z., Diaz-Godinez C., Mora N., Aleman O.R., Uribe-Querol E., Carrero J.C., Rosales C. (2018). Entamoeba histolytica Induce Signaling via Raf/MEK/ERK for Neutrophil Extracellular Trap (NET) Formation. Front. Cell Infect. Microbiol..

[25] Palmer L.J., Cooper P.R., Ling M.R., Wright H.J., Huissoon A., Chapple I.L. (2012). Hypochlorous acid regulates neutrophil extracellular trap release in humans. Clin. Exp. Immunol..

[26] Rohrbach A.S., Slade D.J., Thompson P.R., Mowen K.A. (2012). Activation of PAD4 in NET formation. Front. Immunol..

[27] Vorobjeva N.V., Chernyak B.V. (2020). NETosis: Molecular Mechanisms, Role in Physiology and Pathology. Biochemistry.

[28] Klopf J., Brostjan C., Eilenberg W., Neumayer C. (2021). Neutrophil Extracellular Traps and Their Implications in Cardiovascular and Inflammatory Disease. Int. J. Mol. Sci..

[29] Suliman H.B., Welty-Wolf K.E., Carraway M.S., Schwartz D.A., Hollingsworth J.W., Piantadosi C.A. (2005). Toll-like receptor 4 mediates mitochondrial DNA damage and biogenic responses after heat-inactivated *E. coli*. FASEB J..

[30] Frizinsky S., Haj-Yahia S., Machnes Maayan D., Lifshitz Y., Maoz-Segal R., Offengenden I., Kidon M., Agmon-Levin N. (2019). The innate immune perspective of autoimmune and autoinflammatory conditions. Rheumatology.

[31] Tsourouktsoglou T.D., Warnatsch A., Ioannou M., Hoving D., Wang Q., Papayannopoulos V. (2020). Histones, DNA, and Citrullination Promote Neutrophil Extracellular Trap Inflammation by Regulating the Localization and Activation of TLR4. Cell Rep..

[32] Fatemi A., Alipour R., Khanahmad H., Alsahebfosul F., Andalib A., Pourazar A. (2021). The impact of neutrophil extracellular trap from patients with systemic lupus erythematosus on the viability, CD11b expression and oxidative burst of healthy neutrophils. BMC Immunol..

[33] Wang H., Li T., Chen S., Gu Y., Ye S. (2015). Neutrophil Extracellular Trap Mitochondrial DNA and Its Autoantibody in Systemic Lupus Erythematosus and a Proof-of-Concept Trial of Metformin. Arthritis Rheumatol..

[34] Bach M., Moon J., Moore R., Pan T., Nelson J.L., Lood C. (2020). A Neutrophil Activation Biomarker Panel in Prognosis and Monitoring of Patients with Rheumatoid Arthritis. Arthritis Rheumatol..

[35] Angelidou I., Chrysanthopoulou A., Mitsios A., Arelaki S., Arampatzioglou A., Kambas K., Ritis D., Tsironidou V., Moschos I., Dalla V. (2018). REDD1/Autophagy Pathway Is Associated with Neutrophil-Driven IL-1beta Inflammatory Response in Active Ulcerative Colitis. J. Immunol..

[36] Zhang L., Yuan Y., Xu Q., Jiang Z., Chu C.Q. (2019). Contribution of neutrophils in the pathogenesis of rheumatoid arthritis. J. Biomed. Res..

[37] Khandpur R., Carmona-Rivera C., Vivekanandan-Giri A., Gizinski A., Yalavarthi S., Knight J.S., Friday S., Li S., Patel R.M., Subramanian V. (2013). NETs are a source of citrullinated autoantigens and stimulate inflammatory responses in rheumatoid arthritis. Sci. Transl. Med..

[38] Demoruelle M.K., Harrall K.K., Ho L., Purmalek M.M., Seto N.L., Rothfuss H.M., Weisman M.H., Solomon J.J., Fischer A., Okamoto Y. (2017). Anti-Citrullinated Protein Antibodies Are Associated With Neutrophil Extracellular Traps in the Sputum in Relatives of Rheumatoid Arthritis Patients. Arthritis Rheumatol..

[39] Gestermann N., Di Domizio J., Lande R., Demaria O., Frasca L., Feldmeyer L., Di Lucca J., Gilliet M. (2018). Netting Neutrophils Activate Autoreactive B Cells in Lupus. J. Immunol..

[40] Shi L., Yao H., Liu Z., Xu M., Tsung A., Wang Y. (2020). Endogenous PAD4 in Breast Cancer Cells Mediates Cancer Extracellular Chromatin Network Formation and Promotes Lung Metastasis. Mol. Cancer Res..

[41] Liu Y., Lightfoot Y.L., Seto N., Carmona-Rivera C., Moore E., Goel R., O’Neil L., Mistry P., Hoffmann V., Mondal S. (2018). Peptidylarginine deiminases 2 and 4 modulate innate and adaptive immune responses in TLR-7-dependent lupus. JCI Insight.

[42] Leshner M., Wang S., Lewis C., Zheng H., Chen X.A., Santy L., Wang Y. (2012). PAD4 mediated histone hypercitrullination induces heterochromatin decondensation and chromatin unfolding to form neutrophil extracellular trap-like structures. Front. Immunol..

[43] Li P., Li M., Lindberg M.R., Kennett M.J., Xiong N., Wang Y. (2010). PAD4 is essential for antibacterial innate immunity mediated by neutrophil extracellular traps. J. Exp. Med..

[44] Romero V., Fert-Bober J., Nigrovic P.A., Darrah E., Haque U.J., Lee D.M., van Eyk J., Rosen A., Andrade F. (2013). Immune-mediated pore-forming pathways induce cellular hypercitrullination and generate citrullinated autoantigens in rheumatoid arthritis. Sci. Transl. Med..

[45] Tian Y., Qu S., Alam H.B., Williams A.M., Wu Z., Deng Q., Pan B., Zhou J., Liu B., Duan X. (2020). Peptidylarginine deiminase 2 has potential as both a biomarker and therapeutic target of sepsis. JCI Insight.

[46] Li M., Lin C., Deng H., Strnad J., Bernabei L., Vogl D.T., Burke J.J., Nefedova Y. (2020). A Novel Peptidylarginine Deiminase 4 (PAD4) Inhibitor BMS-P5 Blocks Formation of Neutrophil Extracellular Traps and Delays Progression of Multiple Myeloma. Mol. Cancer Ther..

[47] Suzuki M., Ikari J., Anazawa R., Tanaka N., Katsumata Y., Shimada A., Suzuki E., Tatsumi K. (2020). PAD4 Deficiency Improves Bleomycin-induced Neutrophil Extracellular Traps and Fibrosis in Mouse Lung. Am. J. Respir. Cell Mol. Biol..

[48] Lewis H.D., Liddle J., Coote J.E., Atkinson S.J., Barker M.D., Bax B.D., Bicker K.L., Bingham R.P., Campbell M., Chen Y.H. (2015). Inhibition of PAD4 activity is sufficient to disrupt mouse and human NET formation. Nat. Chem. Biol..

[49] Wu Z., Deng Q., Pan B., Alam H.B., Tian Y., Bhatti U.F., Liu B., Mondal S., Thompson P.R., Li Y. (2020). Inhibition of PAD2 Improves Survival in a Mouse Model of Lethal LPS-Induced Endotoxic Shock. Inflammation.

[50] Korkmaz B., Horwitz M.S., Jenne D.E., Gauthier F. (2010). Neutrophil elastase, proteinase 3, and cathepsin G as therapeutic targets in human diseases. Pharmacol. Rev..

[51] Martinod K., Witsch T., Farley K., Gallant M., Remold-O’Donnell E., Wagner D.D. (2016). Neutrophil elastase-deficient mice form neutrophil extracellular traps in an experimental model of deep vein thrombosis. J. Thromb. Haemost..

[52] Dunlevy F.K., Martin S.L., de Courcey F., Elborn J.S., Ennis M. (2012). Anti-inflammatory effects of DX-890, a human neutrophil elastase inhibitor. J. Cyst. Fibros..

[53] Sullivan A.L., Dafforn T., Hiemstra P.S., Stockley R.A. (2008). Neutrophil elastase reduces secretion of secretory leukoproteinase inhibitor (SLPI) by lung epithelial cells: Role of charge of the proteinase-inhibitor complex. Respir. Res..

[54] Governa V., Trella E., Mele V., Tornillo L., Amicarella F., Cremonesi E., Muraro M.G., Xu H., Droeser R., Daster S.R. (2017). The Interplay Between Neutrophils and CD8(+) T Cells Improves Survival in Human Colorectal Cancer. Clin. Cancer Res..

[55] Vlkova M., Chovancova Z., Nechvatalova J., Connelly A.N., Davis M.D., Slanina P., Travnickova L., Litzman M., Grymova T., Soucek P. (2019). Neutrophil and Granulocytic Myeloid-Derived Suppressor Cell-Mediated T Cell Suppression Significantly Contributes to Immune Dysregulation in Common Variable Immunodeficiency Disorders. J. Immunol..

[56] Cerutti A., Puga I., Magri G. (2013). The B cell helper side of neutrophils. J. Leukoc. Biol..

[57] Puga I., Cols M., Barra C.M., He B., Cassis L., Gentile M., Comerma L., Chorny A., Shan M., Xu W. (2011). B cell-helper neutrophils stimulate the diversification and production of immunoglobulin in the marginal zone of the spleen. Nat. Immunol..

[58] Hosoki K., Itazawa T., Boldogh I., Sur S. (2016). Neutrophil recruitment by allergens contribute to allergic sensitization and allergic inflammation. Curr. Opin. Allergy Clin. Immunol..

[59] Arebro J., Ekstedt S., Hjalmarsson E., Winqvist O., Kumlien Georen S., Cardell L.O. (2017). A possible role for neutrophils in allergic rhinitis revealed after cellular subclassification. Sci. Rep..

[60] Polak D., Hafner C., Briza P., Kitzmuller C., Elbe-Burger A., Samadi N., Gschwandtner M., Pfutzner W., Zlabinger G.J., Jahn-Schmid B. (2019). A novel role for neutrophils in IgE-mediated allergy: Evidence for antigen presentation in late-phase reactions. J. Allergy Clin. Immunol..

[61] Jones N.D., Brook M.O., Carvalho-Gaspar M., Luo S., Wood K.J. (2010). Regulatory T cells can prevent memory CD8+ T-cell-mediated rejection following polymorphonuclear cell depletion. Eur. J. Immunol..

[62] Yildiz M.B., Yildiz E. (2021). Evaluation of serum neutrophil-to-lymphocyte ratio in corneal graft rejection after low-risk penetrating keratoplasty. Int. Ophthalmol..

[63] Liu Y., Qin X., Lei Z., Chai H., Wu Z. (2021). Diphenyleneiodonium ameliorates acute liver rejection during transplantation by inhibiting neutrophil extracellular traps formation in vivo. Transpl. Immunol..

[64] Glenn J.W., Cody M.J., McManus M.P., Pulsipher M.A., Schiffman J.D., Yost C.C. (2016). Deficient Neutrophil Extracellular Trap Formation in Patients Undergoing Bone Marrow Transplantation. Front. Immunol..

[65] Harwood N.E., Barral P., Batista F.D. (2012). Neutrophils—The unexpected helpers of B-cell activation. EMBO Rep..

[66] Oehler L., Majdic O., Pickl W.F., Stockl J., Riedl E., Drach J., Rappersberger K., Geissler K., Knapp W. (1998). Neutrophil granulocyte-committed cells can be driven to acquire dendritic cell characteristics. J. Exp. Med..

[67] Parackova Z., Zentsova I., Vrabcova P., Klocperk A., Sumnik Z., Pruhova S., Petruzelkova L., Hasler R., Sediva A. (2020). Neutrophil Extracellular Trap Induced Dendritic Cell Activation Leads to Th1 Polarization in Type 1 Diabetes. Front. Immunol..

[68] Scapini P., Cassatella M.A. (2014). Social networking of human neutrophils within the immune system. Blood.

[69] Li Y., Wang W., Yang F., Xu Y., Feng C., Zhao Y. (2019). The regulatory roles of neutrophils in adaptive immunity. Cell Commun. Signal..

[70] Yang C.W., Strong B.S., Miller M.J., Unanue E.R. (2010). Neutrophils influence the level of antigen presentation during the immune response to protein antigens in adjuvants. J. Immunol..

[71] Guc E., Pollard J.W. (2021). Redefining macrophage and neutrophil biology in the metastatic cascade. Immunity.

[72] Tillack K., Breiden P., Martin R., Sospedra M. (2012). T lymphocyte priming by neutrophil extracellular traps links innate and adaptive immune responses. J. Immunol..

[73] Yajuk O., Baron M., Toker S., Zelter T., Fainsod-Levi T., Granot Z. (2021). The PD-L1/PD-1 Axis Blocks Neutrophil Cytotoxicity in Cancer. Cells.

[74] Farah C.S., Elahi S., Pang G., Gotjamanos T., Seymour G.J., Clancy R.L., Ashman R.B. (2001). T cells augment monocyte and neutrophil function in host resistance against oropharyngeal candidiasis. Infect. Immun..

[75] Kalyan S., Kabelitz D. (2013). Defining the nature of human gammadelta T cells: A biographical sketch of the highly empathetic. Cell. Mol. Immunol..

[76] Boonyaratanakornkit J., Taylor J.J. (2019). Techniques to Study Antigen-Specific B Cell Responses. Front. Immunol..

[77] Chen K., Xu W., Wilson M., He B., Miller N.W., Bengten E., Edholm E.S., Santini P.A., Rath P., Chiu A. (2009). Immunoglobulin D enhances immune surveillance by activating antimicrobial, proinflammatory and B cell-stimulating programs in basophils. Nat. Immunol..

[78] Van Kessel K.P., Bestebroer J., van Strijp J.A. (2014). Neutrophil-Mediated Phagocytosis of Staphylococcus aureus. Front. Immunol..

[79] Lo L.W., Chang C.W., Chiang M.F., Lin I.Y., Lin K.I. (2021). Marginal Zone B Cells Assist With Neutrophil Accumulation to Fight Against Systemic Staphylococcus aureus Infection. Front. Immunol..

[80] Schofield Z.V., Woodruff T.M., Halai R., Wu M.C., Cooper M.A. (2013). Neutrophils—A key component of ischemia-reperfusion injury. Shock.

[81] Chun Y., Kim J. (2018). Autophagy: An Essential Degradation Program for Cellular Homeostasis and Life. Cells.

[82] Fraiberg M., Elazar Z. (2020). Genetic defects of autophagy linked to disease. Prog. Mol. Biol. Transl. Sci..

[83] Yang Z., Klionsky D.J. (2009). An overview of the molecular mechanism of autophagy. Curr. Top. Microbiol. Immunol..

[84] Qian M., Fang X., Wang X. (2017). Autophagy and inflammation. Clin. Transl. Med..

[85] Yu Y., Sun B. (2020). Autophagy-mediated regulation of neutrophils and clinical applications. Burns Trauma.

[86] Itakura A., McCarty O.J. (2013). Pivotal role for the mTOR pathway in the formation of neutrophil extracellular traps via regulation of autophagy. Am. J. Physiol. Cell Physiol..

[87] Shrestha S., Lee J.M., Hong C.W. (2020). Autophagy in neutrophils. Korean J. Physiol. Pharmacol..

[88] Zhou Z.W., Li X.X., He Z.X., Pan S.T., Yang Y., Zhang X., Chow K., Yang T., Qiu J.X., Zhou Q. (2015). Induction of apoptosis and autophagy via sirtuin1- and PI3K/Akt/mTOR-mediated pathways by plumbagin in human prostate cancer cells. Drug Des. Dev. Ther..

[89] Mroczek A., Cieloch A., Manda-Handzlik A., Kuzmicka W., Muchowicz A., Wachowska M. (2020). Overexpression of ATG5 Gene Makes Granulocyte-Like HL-60 Susceptible to Release Reactive Oxygen Species. Int. J. Mol. Sci..

[90] Xu F., Zhang C., Zou Z., Fan E.K.Y., Chen L., Li Y., Billiar T.R., Wilson M.A., Shi X., Fan J. (2017). Aging-related Atg5 defect impairs neutrophil extracellular traps formation. Immunology.

[91] Bhattacharya A., Wei Q., Shin J.N., Abdel Fattah E., Bonilla D.L., Xiang Q., Eissa N.T. (2015). Autophagy Is Required for Neutrophil-Mediated Inflammation. Cell Rep..

[92] Rosales C. (2020). Neutrophils at the crossroads of innate and adaptive immunity. J. Leukoc. Biol..

[93] Levine B., Mizushima N., Virgin H.W. (2011). Autophagy in immunity and inflammation. Nature.

[94] Remijsen Q., Vanden Berghe T., Wirawan E., Asselbergh B., Parthoens E., De Rycke R., Noppen S., Delforge M., Willems J., Vandenabeele P. (2011). Neutrophil extracellular trap cell death requires both autophagy and superoxide generation. Cell Res..

[95] Rozman S., Yousefi S., Oberson K., Kaufmann T., Benarafa C., Simon H.U. (2015). The generation of neutrophils in the bone marrow is controlled by autophagy. Cell Death Differ..

[96] Zhang C., Syed T.W., Liu R., Yu J. (2017). Role of Endoplasmic Reticulum Stress, Autophagy, and Inflammation in Cardiovascular Disease. Front. Cardiovasc. Med..

[97] Cho B.J., Hwang J.S., Shin Y.J., Kim J.W., Chung T.Y., Hyon J.Y. (2019). Rapamycin Rescues Endoplasmic Reticulum Stress-Induced Dry Eye Syndrome in Mice. Investig. Ophthalmol. Vis. Sci..

[98] Jin L., Batra S., Jeyaseelan S. (2017). Deletion of Nlrp3 Augments Survival during Polymicrobial Sepsis by Decreasing Autophagy and Enhancing Phagocytosis. J. Immunol..

[99] Rosales C. (2018). Neutrophil: A Cell with Many Roles in Inflammation or Several Cell Types?. Front. Physiol..

[100] Park Y.J., Yoo S.A., Kim W.U. (2014). Role of endoplasmic reticulum stress in rheumatoid arthritis pathogenesis. J. Korean Med. Sci..

[101] Eizirik D.L., Cardozo A.K., Cnop M. (2008). The role for endoplasmic reticulum stress in diabetes mellitus. Endocr. Rev..

[102] Ghosh R., Colon-Negron K., Papa F.R. (2019). Endoplasmic reticulum stress, degeneration of pancreatic islet beta-cells, and therapeutic modulation of the unfolded protein response in diabetes. Mol. Metab..

[103] Santos L.E., Ferreira S.T. (2018). Crosstalk between endoplasmic reticulum stress and brain inflammation in Alzheimer’s disease. Neuropharmacology.

[104] Tanimura A., Miyoshi K., Horiguchi T., Hagita H., Fujisawa K., Noma T. (2018). Mitochondrial Activity and Unfolded Protein Response are Required for Neutrophil Differentiation. Cell. Physiol. Biochem..

[105] Hu R., Chen Z.F., Yan J., Li Q.F., Huang Y., Xu H., Zhang X.P., Jiang H. (2015). Endoplasmic Reticulum Stress of Neutrophils Is Required for Ischemia/Reperfusion-Induced Acute Lung Injury. J. Immunol..

[106] Sule G., Abuaita B.H., Steffes P.A., Fernandes A.T., Estes S.K., Dobry C., Pandian D., Gudjonsson J.E., Kahlenberg J.M., O’Riordan M.X. (2021). Endoplasmic reticulum stress sensor IRE1alpha propels neutrophil hyperactivity in lupus. J. Clin. Investig..

[107] White M.M., Geraghty P., Hayes E., Cox S., Leitch W., Alfawaz B., Lavelle G.M., McElvaney O.J., Flannery R., Keenan J. (2017). Neutrophil Membrane Cholesterol Content is a Key Factor in Cystic Fibrosis Lung Disease. EBioMedicine.

[108] Guo Q., Li H., Liu J., Xu L., Yang L., Sun Z., Zhou B. (2017). Tunicamycin aggravates endoplasmic reticulum stress and airway inflammation via PERK-ATF4-CHOP signaling in a murine model of neutrophilic asthma. J. Asthma.

[109] Xu X., Li Q., Li L., Zeng M., Zhou X., Cheng Z. (2021). Endoplasmic reticulum stress/XBP1 promotes airway mucin secretion under the influence of neutrophil elastase. Int. J. Mol. Med..

[110] Garcia-Navas R., Gajate C., Mollinedo F. (2021). Neutrophils drive endoplasmic reticulum stress-mediated apoptosis in cancer cells through arginase-1 release. Sci. Rep..

[111] Binet F., Cagnone G., Crespo-Garcia S., Hata M., Neault M., Dejda A., Wilson A.M., Buscarlet M., Mawambo G.T., Howard J.P. (2020). Neutrophil extracellular traps target senescent vasculature for tissue remodeling in retinopathy. Science.

[112] Roy-O’Reilly M.A., Ahnstedt H., Spychala M.S., Munshi Y., Aronowski J., Sansing L.H., McCullough L.D. (2020). Aging exacerbates neutrophil pathogenicity in ischemic stroke. Aging.

[113] Weisenburger-Lile D., Dong Y., Yger M., Weisenburger G., Polara G.F., Chaigneau T., Ochoa R.Z., Marro B., Lapergue B., Alamowitch S. (2019). Harmful neutrophil subsets in patients with ischemic stroke: Association with disease severity. Neurol. Neuroimmunol. Neuroinflamm..

[114] Zhang D., Chen G., Manwani D., Mortha A., Xu C., Faith J.J., Burk R.D., Kunisaki Y., Jang J.E., Scheiermann C. (2015). Neutrophil ageing is regulated by the microbiome. Nature.

[115] Zhang D., Frenette P.S. (2019). Cross talk between neutrophils and the microbiota. Blood.

[116] Mangold A., Alias S., Scherz T., Hofbauer M., Jakowitsch J., Panzenbock A., Simon D., Laimer D., Bangert C., Kammerlander A. (2015). Coronary neutrophil extracellular trap burden and deoxyribonuclease activity in ST-elevation acute coronary syndrome are predictors of ST-segment resolution and infarct size. Circ. Res..

[117] Uhl B., Vadlau Y., Zuchtriegel G., Nekolla K., Sharaf K., Gaertner F., Massberg S., Krombach F., Reichel C.A. (2016). Aged neutrophils contribute to the first line of defense in the acute inflammatory response. Blood.

[118] Kang L., Yu H., Yang X., Zhu Y., Bai X., Wang R., Cao Y., Xu H., Luo H., Lu L. (2020). Neutrophil extracellular traps released by neutrophils impair revascularization and vascular remodeling after stroke. Nat. Commun..

[119] Hakkim A., Furnrohr B.G., Amann K., Laube B., Abed U.A., Brinkmann V., Herrmann M., Voll R.E., Zychlinsky A. (2010). Impairment of neutrophil extracellular trap degradation is associated with lupus nephritis. Proc. Natl. Acad. Sci. USA.

[120] Tulgar Y.K., Cakar S., Tulgar S., Dalkilic O., Cakiroglu B., Uyanik B.S. (2016). The effect of smoking on neutrophil/lymphocyte and platelet/lymphocyte ratio and platelet indices: A retrospective study. Eur. Rev. Med. Pharmacol. Sci..

[121] Hoonhorst S.J., Timens W., Koenderman L., Lo Tam Loi A.T., Lammers J.W., Boezen H.M., van Oosterhout A.J., Postma D.S., Ten Hacken N.H. (2014). Increased activation of blood neutrophils after cigarette smoking in young individuals susceptible to COPD. Respir. Res..

[122] Lee K.H., Lee J., Jeong J., Woo J., Lee C.H., Yoo C.G. (2018). Cigarette smoke extract enhances neutrophil elastase-induced IL-8 production via proteinase-activated receptor-2 upregulation in human bronchial epithelial cells. Exp. Mol. Med..

[123] Mortaz E., Adcock I.M., Ito K., Kraneveld A.D., Nijkamp F.P., Folkerts G. (2010). Cigarette smoke induces CXCL8 production by human neutrophils via activation of TLR9 receptor. Eur. Respir. J..

[124] Xu X., Jiang S.Y., Wang T.Y., Bai Y., Zhong M., Wang A., Lippmann M., Chen L.C., Rajagopalan S., Sun Q. (2013). Inflammatory response to fine particulate air pollution exposure: Neutrophil versus monocyte. PLoS ONE.

[125] Jeong S., Park S.A., Park I., Kim P., Cho N.H., Hyun J.W., Hyun Y.M. (2019). PM2.5 Exposure in the Respiratory System Induces Distinct Inflammatory Signaling in the Lung and the Liver of Mice. J. Immunol. Res..

[126] Zhang Y., Geng S., Prasad G.L., Li L. (2018). Suppression of Neutrophil Antimicrobial Functions by Total Particulate Matter From Cigarette Smoke. Front. Immunol..

[127] Sato J., Takahashi I., Umeda T., Matsuzaka M., Danjyo K., Tsuya R., Kida K., Takami H., Nakaji S. (2011). Effect of alcohol drinking and cigarette smoking on neutrophil functions in adults. Luminescence.

[128] Bukong T.N., Cho Y., Iracheta-Vellve A., Saha B., Lowe P., Adejumo A., Furi I., Ambade A., Gyongyosi B., Catalano D. (2018). Abnormal neutrophil traps and impaired efferocytosis contribute to liver injury and sepsis severity after binge alcohol use. J. Hepatol..

[129] Stadlbauer V., Horvath A., Komarova I., Schmerboeck B., Feldbacher N., Wurm S., Klymiuk I., Durdevic M., Rainer F., Blesl A. (2019). A single alcohol binge impacts on neutrophil function without changes in gut barrier function and gut microbiome composition in healthy volunteers. PLoS ONE.

[130] Malacco N., Souza J.A.M., Martins F.R.B., Rachid M.A., Simplicio J.A., Tirapelli C.R., Sabino A.P., Queiroz-Junior C.M., Goes G.R., Vieira L.Q. (2020). Chronic ethanol consumption compromises neutrophil function in acute pulmonary Aspergillus fumigatus infection. eLife.

[131] Subramanian K., Bergman P., Henriques-Normark B. (2017). Vitamin D Promotes Pneumococcal Killing and Modulates Inflammatory Responses in Primary Human Neutrophils. J. Innate Immun..

[132] Chen L., Eapen M.S., Zosky G.R. (2017). Vitamin D both facilitates and attenuates the cellular response to lipopolysaccharide. Sci. Rep..

[133] Wang S.Y., Shen T.T., Xi B.L., Shen Z., Zhang X. (2021). Vitamin D affects the neutrophil-to-lymphocyte ratio in patients with type 2 diabetes mellitus. J. Diabetes Investig..

[134] Machado Cda S., Venancio V.P., Aissa A.F., Hernandes L.C., de Mello M.B., Del Lama J.E., Marzocchi-Machado C.M., Bianchi M.L., Antunes L.M. (2016). Vitamin D3 deficiency increases DNA damage and the oxidative burst of neutrophils in a hypertensive rat model. Mutat. Res. Genet. Toxicol. Environ. Mutagen..

[135] Zhao L., Xu T., Li Y., Luan Y., Lv Q., Fu G., Zhang W. (2020). Variability in blood lipids affects the neutrophil to lymphocyte ratio in patients undergoing elective percutaneous coronary intervention: A retrospective study. Lipids Health Dis..

[136] Lai X.F., Qin H.D., Guo L.L., Luo Z.G., Chang J., Qin C.C. (2014). Hypercholesterolemia increases the production of leukotriene B4 in neutrophils by enhancing the nuclear localization of 5-lipoxygenase. Cell. Physiol. Biochem..

[137] Drechsler M., Megens R.T., van Zandvoort M., Weber C., Soehnlein O. (2010). Hyperlipidemia-triggered neutrophilia promotes early atherosclerosis. Circulation.

[138] Bosco A.M., de Almeida B.F., Pereira P.P., Narciso L.G., Lima V.M., Ciarlini P.C. (2013). High concentrations of glucose reduce the oxidative metabolism of dog neutrophils in vitro. BMC Vet. Res..

[139] Kummer U., Zobeley J., Brasen J.C., Fahmy R., Kindzelskii A.L., Petty A.R., Clark A.J., Petty H.R. (2007). Elevated glucose concentrations promote receptor-independent activation of adherent human neutrophils: An experimental and computational approach. Biophys. J..

[140] Wong S.L., Demers M., Martinod K., Gallant M., Wang Y., Goldfine A.B., Kahn C.R., Wagner D.D. (2015). Diabetes primes neutrophils to undergo NETosis, which impairs wound healing. Nat. Med..

[141] Jiang L., Tang K., Levin M., Irfan O., Morris S.K., Wilson K., Klein J.D., Bhutta Z.A. (2020). COVID-19 and multisystem inflammatory syndrome in children and adolescents. Lancet Infect. Dis..

[142] V’Kovski P., Kratzel A., Steiner S., Stalder H., Thiel V. (2021). Coronavirus biology and replication: Implications for SARS-CoV-2. Nat. Rev. Microbiol..

[143] Bansal M. (2020). Cardiovascular disease and COVID-19. Diabetes Metab. Syndr..

[144] Leppkes M., Knopf J., Naschberger E., Lindemann A., Singh J., Herrmann I., Sturzl M., Staats L., Mahajan A., Schauer C. (2020). Vascular occlusion by neutrophil extracellular traps in COVID-19. EBioMedicine.

[145] Radermecker C., Detrembleur N., Guiot J., Cavalier E., Henket M., d’Emal C., Vanwinge C., Cataldo D., Oury C., Delvenne P. (2020). Neutrophil extracellular traps infiltrate the lung airway, interstitial, and vascular compartments in severe COVID-19. J. Exp. Med..

[146] Veras F.P., Pontelli M.C., Silva C.M., Toller-Kawahisa J.E., de Lima M., Nascimento D.C., Schneider A.H., Caetite D., Tavares L.A., Paiva I.M. (2020). SARS-CoV-2-triggered neutrophil extracellular traps mediate COVID-19 pathology. J. Exp. Med..

[147] Middleton E.A., He X.Y., Denorme F., Campbell R.A., Ng D., Salvatore S.P., Mostyka M., Baxter-Stoltzfus A., Borczuk A.C., Loda M. (2020). Neutrophil extracellular traps contribute to immunothrombosis in COVID-19 acute respiratory distress syndrome. Blood.

[148] Folco E.J., Mawson T.L., Vromman A., Bernardes-Souza B., Franck G., Persson O., Nakamura M., Newton G., Luscinskas F.W., Libby P. (2018). Neutrophil Extracellular Traps Induce Endothelial Cell Activation and Tissue Factor Production Through Interleukin-1alpha and Cathepsin G. Arterioscler. Thromb. Vasc. Biol..

[149] Skendros P., Mitsios A., Chrysanthopoulou A., Mastellos D.C., Metallidis S., Rafailidis P., Ntinopoulou M., Sertaridou E., Tsironidou V., Tsigalou C. (2020). Complement and tissue factor-enriched neutrophil extracellular traps are key drivers in COVID-19 immunothrombosis. J. Clin. Investig..

[150] Fisher J., Mohanty T., Karlsson C.A.Q., Khademi S.M.H., Malmstrom E., Frigyesi A., Nordenfelt P., Malmstrom J., Linder A. (2021). Proteome Profiling of Recombinant DNase Therapy in Reducing NETs and Aiding Recovery in COVID-19 Patients. Mol. Cell. Proteom..

[151] Zuo Y., Yalavarthi S., Shi H., Gockman K., Zuo M., Madison J.A., Blair C., Weber A., Barnes B.J., Egeblad M. (2020). Neutrophil extracellular traps (NETs) as markers of disease severity in COVID-19. medRxiv.

[152] Lee Y.Y., Park H.H., Park W., Kim H., Jang J.G., Hong K.S., Lee J.Y., Seo H.S., Na D.H., Kim T.H. (2021). Long-acting nanoparticulate DNase-1 for effective suppression of SARS-CoV-2-mediated neutrophil activities and cytokine storm. Biomaterials.

[153] Thanabalasuriar A., Scott B.N.V., Peiseler M., Willson M.E., Zeng Z., Warrener P., Keller A.E., Surewaard B.G.J., Dozier E.A., Korhonen J.T. (2019). Neutrophil Extracellular Traps Confine Pseudomonas aeruginosa Ocular Biofilms and Restrict Brain Invasion. Cell Host Microbe.

[154] Clark H.L., Abbondante S., Minns M.S., Greenberg E.N., Sun Y., Pearlman E. (2018). Protein Deiminase 4 and CR3 Regulate Aspergillus fumigatus and beta-Glucan-Induced Neutrophil Extracellular Trap Formation, but Hyphal Killing Is Dependent Only on CR3. Front. Immunol..

[155] Kandhavelu J., Demonte N.L., Namperumalsamy V.P., Prajna L., Thangavel C., Jayapal J.M., Kuppamuthu D. (2016). Data set of Aspergillus flavus induced alterations in tear proteome: Understanding the pathogen-induced host response to fungal infection. Data Brief.

[156] Lappann M., Danhof S., Guenther F., Olivares-Florez S., Mordhorst I.L., Vogel U. (2013). In vitro resistance mechanisms of Neisseria meningitidis against neutrophil extracellular traps. Mol. Microbiol..

[157] Pilsczek F.H., Salina D., Poon K.K., Fahey C., Yipp B.G., Sibley C.D., Robbins S.M., Green F.H., Surette M.G., Sugai M. (2010). A novel mechanism of rapid nuclear neutrophil extracellular trap formation in response to Staphylococcus aureus. J. Immunol..

[158] Berends E.T., Horswill A.R., Haste N.M., Monestier M., Nizet V., von Kockritz-Blickwede M. (2010). Nuclease expression by Staphylococcus aureus facilitates escape from neutrophil extracellular traps. J. Innate Immun..

[159] Shan Q., Dwyer M., Rahman S., Gadjeva M. (2014). Distinct susceptibilities of corneal Pseudomonas aeruginosa clinical isolates to neutrophil extracellular trap-mediated immunity. Infect. Immun..

[160] Zhu B., Zhang L., Yuan K., Huang X., Hu R., Jin X. (2021). Neutrophil extracellular traps may have a dual role in Pseudomonas aeruginosa keratitis. Eur. J. Clin. Microbiol. Infect. Dis..

[161] Fan F., Huang X., Yuan K., Zhu B., Zhao Y., Hu R., Wan T., Zhu L., Jin X. (2020). Glucocorticoids May Exacerbate Fungal Keratitis by Increasing Fungal Aggressivity and Inhibiting the Formation of Neutrophil Extracellular Traps. Curr. Eye Res..

[162] Jin X., Zhao Y., Zhang F., Wan T., Fan F., Xie X., Lin Z. (2016). Neutrophil extracellular traps involvement in corneal fungal infection. Mol. Vis..

[163] Azher T.N., Yin X.T., Stuart P.M. (2017). Understanding the Role of Chemokines and Cytokines in Experimental Models of Herpes Simplex Keratitis. J. Immunol. Res.

[164] Shen F.H., Wang S.W., Yeh T.M., Tung Y.Y., Hsu S.M., Chen S.H. (2013). Absence of CXCL10 aggravates herpes stromal keratitis with reduced primary neutrophil influx in mice. J. Virol..

[165] Chintakuntlawar A.V., Chodosh J. (2009). Chemokine CXCL1/KC and its receptor CXCR2 are responsible for neutrophil chemotaxis in adenoviral keratitis. J. Interferon Cytokine Res..

[166] Molesworth-Kenyon S.J., Yin R., Oakes J.E., Lausch R.N. (2008). IL-17 receptor signaling influences virus-induced corneal inflammation. J. Leukoc. Biol..

[167] Suryawanshi A., Veiga-Parga T., Rajasagi N.K., Reddy P.B., Sehrawat S., Sharma S., Rouse B.T. (2011). Role of IL-17 and Th17 cells in herpes simplex virus-induced corneal immunopathology. J. Immunol..

[168] Eslani M., Baradaran-Rafii A., Movahedan A., Djalilian A.R. (2014). The ocular surface chemical burns. J. Ophthalmol..

[169] Baradaran-Rafii A., Eslani M., Haq Z., Shirzadeh E., Huvard M.J., Djalilian A.R. (2017). Current and Upcoming Therapies for Ocular Surface Chemical Injuries. Ocul. Surf..

[170] Wan T., Zhang Y., Yuan K., Min J., Mou Y., Jin X. (2020). Acetylsalicylic Acid Promotes Corneal Epithelium Migration by Regulating Neutrophil Extracellular Traps in Alkali Burn. Front. Immunol..

[171] Shimazaki J. (2018). Definition and Diagnostic Criteria of Dry Eye Disease: Historical Overview and Future Directions. Investig. Opthalmology Vis. Sci..

[172] Postnikoff C.K., Held K., Viswanath V., Nichols K.K. (2020). Enhanced closed eye neutrophil degranulation in dry eye disease. Ocul. Surf..

[173] Gorbet M., Postnikoff C., Williams S. (2015). The Noninflammatory Phenotype of Neutrophils From the Closed-Eye Environment: A Flow Cytometry Analysis of Receptor Expression. Investig. Opthalmology Vis. Sci..

[174] Barliya T., Dardik R., Nisgav Y., Dachbash M., Gaton D., Kenet G., Ehrlich R., Weinberger D., Livnat T. (2017). Possible involvement of NETosis in inflammatory processes in the eye: Evidence from a small cohort of patients. Mol. Vis..

[175] Sonawane S., Khanolkar V., Namavari A., Chaudhary S., Gandhi S., Tibrewal S., Jassim S.H., Shaheen B., Hallak J., Horner J.H. (2012). Ocular surface extracellular DNA and nuclease activity imbalance: A new paradigm for inflammation in dry eye disease. Investig. Opthalmology Vis. Sci..

[176] Tibrewal S., Ivanir Y., Sarkar J., Nayeb-Hashemi N., Bouchard C.S., Kim E., Jain S. (2014). Hyperosmolar stress induces neutrophil extracellular trap formation: Implications for dry eye disease. Investig. Opthalmology Vis. Sci..

[177] Geerling G., Baudouin C., Aragona P., Rolando M., Boboridis K.G., Benitez-Del-Castillo J.M., Akova Y.A., Merayo-Lloves J., Labetoulle M., Steinhoff M. (2017). Emerging strategies for the diagnosis and treatment of meibomian gland dysfunction: Proceedings of the OCEAN group meeting. Ocul. Surf..

[178] Leppkes M., Maueroder C., Hirth S., Nowecki S., Gunther C., Billmeier U., Paulus S., Biermann M., Munoz L.E., Hoffmann M. (2016). Externalized decondensed neutrophil chromatin occludes pancreatic ducts and drives pancreatitis. Nat. Commun..

[179] Mahajan A., Hasikova L., Hampel U., Gruneboom A., Shan X., Herrmann I., Garreis F., Bock F., Knopf J., Singh J. (2021). Aggregated neutrophil extracellular traps occlude Meibomian glands during ocular surface inflammation. Ocul. Surf..

[180] Ozarslan Ozcan D., Kurtul B.E., Ozcan S.C., Elbeyli A. (2020). Increased Systemic Immune-Inflammation Index Levels in Patients with Dry Eye Disease. Ocul. Immunol. Inflamm..

[181] Targonska-Stepniak B., Zwolak R., Piotrowski M., Grzechnik K., Majdan M. (2020). The Relationship between Hematological Markers of Systemic Inflammation (Neutrophil-To-Lymphocyte, Platelet-To-Lymphocyte, Lymphocyte-To-Monocyte Ratios) and Ultrasound Disease Activity Parameters in Patients with Rheumatoid Arthritis. J. Clin. Med..

[182] Cao X., Zhao M., Li H., Xu D., Li M., Zhang X., Zhang F., Hou Y., Zeng X. (2021). Three new inflammatory markers C reactive protein to albumin ratio, neutrophil to lymphocyte ratio, and platelet to lymphocyte ratio correlated with relapsing polychondritis disease activity index. Clin. Rheumatol..

[183] Sekeryapan B., Uzun F., Buyuktarakci S., Bulut A., Oner V. (2016). Neutrophil-to-Lymphocyte Ratio Increases in Patients With Dry Eye. Cornea.

[184] Balta S., Ozturk C. (2015). The platelet-lymphocyte ratio: A simple, inexpensive and rapid prognostic marker for cardiovascular events. Platelets.

[185] Barden A.E., Shinde S., Burke V., Puddey I.B., Beilin L.J., Irish A.B., Watts G.F., Mori T.A. (2018). The effect of n-3 fatty acids and coenzyme Q10 supplementation on neutrophil leukotrienes, mediators of inflammation resolution and myeloperoxidase in chronic kidney disease. Prostaglandins Other Lipid Mediat..

[186] Gao Y., Su J., Zhang Y., Chan A., Sin J.H., Wu D., Min K., Gronert K. (2018). Dietary DHA amplifies LXA4 circuits in tissues and lymph node PMN and is protective in immune-driven dry eye disease. Mucosal. Immunol..

[187] Hayashi T. (2011). Dysfunction of lacrimal and salivary glands in Sjogren’s syndrome: Nonimmunologic injury in preinflammatory phase and mouse model. J. Biomed. Biotechnol..

[188] Mavragani C.P., Tzioufas A.G., Moutsopoulos H.M. (2000). Sjogren’s syndrome: Autoantibodies to cellular antigens. Clinical and molecular aspects. Int. Arch. Allergy Immunol..

[189] Wu C.H., Li K.J., Yu C.L., Tsai C.Y., Hsieh S.C. (2015). Sjogren’s Syndrome Antigen B Acts as an Endogenous Danger Molecule to Induce Interleukin-8 Gene Expression in Polymorphonuclear Neutrophils. PLoS ONE.

[190] An S., Raju I., Surenkhuu B., Kwon J.E., Gulati S., Karaman M., Pradeep A., Sinha S., Mun C., Jain S. (2019). Neutrophil extracellular traps (NETs) contribute to pathological changes of ocular graft-vs.-host disease (oGVHD) dry eye: Implications for novel biomarkers and therapeutic strategies. Ocul. Surf..

[191] Nakazawa D., Kudo T. (2021). Novel Therapeutic Strategy Based on Neutrophil Subset and Its Function in Autoimmune Dis.ease. Front. Pharmacol..

[192] Geerts L., Jorens P.G., Willems J., De Ley M., Slegers H. (2001). Natural inhibitors of neutrophil function in acute respiratory distress syndrome. Crit. Care Med..

[193] Sahebnasagh A., Saghafi F., Safdari M., Khataminia M., Sadremomtaz A., Talaei Z., Rezai Ghaleno H., Bagheri M., Habtemariam S., Avan R. (2020). Neutrophil elastase inhibitor (sivelestat) may be a promising therapeutic option for management of acute lung injury/acute respiratory distress syndrome or disseminated intravascular coagulation in COVID-19. J. Clin. Pharm. Ther..

[194] Pu S., Wang D., Liu D., Zhao Y., Qi D., He J., Zhou G. (2017). Effect of sivelestat sodium in patients with acute lung injury or acute respiratory distress syndrome: A meta-analysis of randomized controlled trials. BMC Pulm. Med..

[195] Fujii M., Bessho R. (2020). Neutrophil Elastase Inhibitor Sivelestat Attenuates Myocardial Injury after Cardioplegic Arrest in Rat Hearts. Ann. Thorac. Cardiovasc. Surg..

[196] Papadopoulos M.C., Verkman A.S. (2012). Aquaporin 4 and neuromyelitis optica. Lancet Neurol..

[197] Ebata R., Yasukawa K., Nagai K., Saito Y., Higashi K., Homma J., Takada N., Takechi F., Saito N., Kobayashi H. (2019). Sivelestat sodium hydrate treatment for refractory Kawasaki disease. Pediatr. Int..

[198] Yu X., Zhao L., Yu Z., Yu C., Bi J., Sun B., Cong H. (2017). Sivelestat sodium hydrate improves post-traumatic knee osteoarthritis through nuclear factor-kappaB in a rat model. Exp. Ther. Med..

[199] Zang S.F., Ma X.J., Wang L., Zhu G.L., Yang W.J., Liu Y.L., Yan J., Luo Y., Zhuang Z.J., Chen J.Y. (2017). Sivelestat alleviates nonalcoholic steatohepatitis in mice through inhibiting activation of Kupffer cells. Zhonghua Gan Zang Bing Za Zhi.

[200] Xiao X.G., Zu H.G., Li Q.G., Huang P. (2016). Sivelestat sodium hydrate attenuates acute lung injury by decreasing systemic inflammation in a rat model of severe burns. Eur. Rev. Med. Pharmacol. Sci..

[201] Watz H., Nagelschmitz J., Kirsten A., Pedersen F., van der Mey D., Schwers S., Bandel T.J., Rabe K.F. (2019). Safety and efficacy of the human neutrophil elastase inhibitor BAY 85-8501 for the treatment of non-cystic fibrosis bronchiectasis: A randomized controlled trial. Pulm. Pharmacol. Ther..

[202] Chen K.J., Chen Y.L., Ueng S.H., Hwang T.L., Kuo L.M., Hsieh P.W. (2021). Neutrophil elastase inhibitor (MPH-966) improves intestinal mucosal damage and gut microbiota in a mouse model of 5-fluorouracil-induced intestinal mucositis. Biomed. Pharmacother..

[203] Liang Y., Pan B., Alam H.B., Deng Q., Wang Y., Chen E., Liu B., Tian Y., Williams A.M., Duan X. (2018). Inhibition of peptidylarginine deiminase alleviates LPS-induced pulmonary dysfunction and improves survival in a mouse model of lethal endotoxemia. Eur. J. Pharmacol..

[204] Zhou Y., An L.L., Chaerkady R., Mittereder N., Clarke L., Cohen T.S., Chen B., Hess S., Sims G.P., Mustelin T. (2018). Evidence for a direct link between PAD4-mediated citrullination and the oxidative burst in human neutrophils. Sci. Rep..

